# Production of Androgens by Microbial Transformation of Progesterone *in Vitro*: A Model for Androgen Production in Rivers Receiving Paper Mill Effluent

**DOI:** 10.1289/ehp.7161

**Published:** 2004-07-22

**Authors:** Ronald L. Jenkins, Elizabeth M. Wilson, Robert A. Angus, W. Mike Howell, Marion Kirk, Ray Moore, Marione Nance, Amber Brown

**Affiliations:** ^1^Department of Biology, Samford University, Birmingham, Alabama, USA; ^2^Laboratories for Reproductive Biology,; ^3^Department of Pediatrics, and; ^4^Department of Biochemistry and Biophysics, University of North Carolina, Chapel Hill, North Carolina, USA; ^5^Biology Department and; ^6^Comprehensive Cancer Center Mass Spectrometry Shared Facility, University of Alabama at Birmingham, Birmingham, Alabama, USA

**Keywords:** 17α-hydroxyprogesterone, androgen-dependent gene expression, androstadienedione, androstenedione, biotransformation of progesterone, environmental androgens, Fenholloway River, Florida, *Gambusia holbrooki*, masculinized mosquitofish, *Mycobacterium smegmatis*

## Abstract

We have previously documented the presence of progesterone and androstenedione in the water column and bottom sediments of the Fenholloway River, Taylor County, Florida. This river receives paper mill effluent and contains masculinized female mosquitofish. We hypothesized that plant sterols (e.g., β-sitosterol) derived from the pulping of pine trees are transformed by bacteria into progesterone and subsequently into 17α-hydroxyprogesterone, androstenedione, and other androgens. In this study, we demonstrate that these same androgens can be produced *in vitro* from the bacterium *Mycobacterium smegmatis*. In a second part to this study, we reextracted and reanalyzed the sediment from the Fenholloway River and verified the presence of androstadienedione, a Δ1 steroid with androgen activity.

The presence of environmental androgens in biologically effective concentrations was first demonstrated > 20 years ago when a population of the eastern mosquitofish, *Gambusia holbrooki*, was discovered in which the females were all masculinized (Howell et al. 1980). Since then, other populations of masculinized mosquitofish have been discovered, all living in small coastal streams in Florida that receive paper mill effluent (Bortone and Cody 1999). The presence of androgens in pulp mill effluent does not appear to be unique to Florida. Larsson et al. (2000) recently reported male-biased sex ratios in embryos of the marine live-bearing eelpout, *Zoarces viviparous*, exposed to paper mill effluent off the coast of Sweden.

In the last 3 years, some of the androgens in a river containing masculinized, paper mill effluent-exposed mosquitofish have been identified. In an earlier study, we used an androgen-dependent gene expression assay to detect, and mass spectrometry to identify, androstenedione (AED) at a concentration of 0.14 nM in the water column of the Fenholloway River (Jenkins et al. 2001). Parks et al. (2001) used a similar androgen-dependent reporter assay and radioimmunoassay to detect an androgenic substance, presumably testosterone, in samples from the Fenholloway River, Taylor County, Florida. In a subsequent study, we (Jenkins et al. 2003) identified higher concentrations of AED (2.4 nM) and its biosynthetic precursor progesterone (155 nM) in sediment samples from one of the sites of the Fenholloway River studied by Parks et al. (2001). Durhan et al. (2002) presented evidence that there are other androgenic compounds, not clearly identified, in the Fenholloway River below the paper mill.

Orlando et al. (2002) noted that the levels of AED in Fenholloway River water did not seem to be sufficient to cause masculinization of the fish, and they hypothesized that the masculinizing effects from paper mill effluent might result from inhibition of the enzyme P450 aromatase. This aromatase is responsible for the biosynthesis of estrogens from androgens such as AED and testosterone (Thompson and Sitteri 1974). If aromatase activity in mosquitofish exposed to paper mill effluent was inhibited, the concentrations of endogenous androgens would increase and potentially cause masculinization. Orlando et al. (2002) showed the aromatase-inactivation hypothesis to be false, finding that brain and ovarian aromatase activity in the mosquitofish in the Fenholloway River was significantly greater than that of controls. The androgenic substances in the Fenholloway River appeared to be increasing aromatase activity in the brain and ovaries of these fish.

After demonstrating the presence of high levels of progesterone in the river sediment, we (Jenkins et al. 2003) hypothesized that some or all of the androgens in paper mill effluent and river sediment were generated by microbial degradation of phytosterols from the mill pulp. To test this hypothesis, in the present study we incubated a common soil bacterium, *Mycobacterium smegmatis*, with progesterone. Androgenic activity of chemicals in the medium was detected using an androgen receptor transcription assay and was quantified by high-pressure liquid chromatography (HPLC). Some of the androgens found in the microbial incubation study were not previously detected in our analyses of sediment from the Fenholloway River (Jenkins et al. 2003). To determine if these previously unidentified substances were indeed present in the Fenholloway River, in the present study, we reextracted and analyzed the sediment using an androgen receptor transcription assay, HPLC, and liquid chromatography mass spectrometry (LC-MS).

## Materials and Methods

### Microbial incubations, extraction, and HPLC separation.

*Mycobacterium smegmatis* (strain ATCC 14468 from Presque Isle Cultures, Erie, PA) were grown in 200 mL Bacto Nutrient broth (3 g beef extract plus 5 g peptone/L; Difco Laboratories, Detroit, MI) at 24–25°C to yield a cell density of 4 × 10^6^/mL. To determine *M. smegmatis* density, we prepared dilution plates using the pour-plate technique, and we used a Quebec colony counter (Fisher Scientific, Atlanta, GA) for viewing and counting the isolated colonies.

Progesterone (P0130; Sigma Chemical Co., St. Louis, MO) was added to each culture to yield a 1-mM solution. Before progesterone was added and on days 0, 2, 4, 6, 8, 12, 15, 20, and 36 after progesterone was added, triplicate 3-mL samples of the slurried media were extracted in 12 mL 100% methanol (HPLC grade). The vortexed extract was centrifuged (1,000 × *g*, 10 min) and passed through a 0.3 mL washed C-18 solid-phase extraction cartridge (Varian Instrument Co., Walnut Creek, CA). The eluant was dried under nitrogen gas before fractionation.

Each extract was fractionated on a 4.6 × 15 cm C-18 column (Varian Instrument Co.) using a 35-min HPLC methanol linear gradient with 0.25% *O*-phosphoric acid and 100% methanol. The gradient increased from 20% methanol at 0 min to 100% methanol at 20–35 min. Ultraviolet detection of column eluants was at 235 nm, and identification was based on co-migration with standards. Quantification of steroids was based on peak area relative to standards, and data are presented as the mean of triplicates (± SE; micromolar) (Jenkins et al. 2003).

### Efficiency of steroid extraction.

The percent recovery of extracted steroids in all bacterial sample fractions was determined by sequentially extracting the samples a second and third time in methanol, followed by solid-phase extraction and quantification by HPLC. The percent yield of progesterone, 17α-hydroxyprogesterone (17α-OHP), AED, and androstadienedione (ADD) at each level was 86.7 ± 2.9 % (mean ± SE).

### Androgen receptor transcription assays.

The agonist activity of compounds separated from bacterial media extracts by HPLC was determined in transient cotransfection assays using a luciferase reporter gene (He et al. 2001; Jenkins et al. 2001; Kemppainen et al. 1992). Briefly, monkey kidney CV-1 cells (0.42 × 10^6^ cells/6-cm dish) were transfected using the calcium phosphate DNA precipitation method with the human androgen receptor expression vector pCMVhAR (50 or 100 ng/dish) and the luciferase reporter vector under control of the mouse mammary tumor virus promoter (MMTV-luc, 5 μg/dish). Cells were incubated for 24 hr at 37°C with dihydrotestosterone (DHT; 0.01, 0.1, or 1 nM concentrations) or 10 μL additions of HPLC fractions. Representative luciferase activity assays are expressed in optical units relative to the no-hormone control. The MMTV-luc assay performed in CV-1 cells demonstrates human androgen receptor–mediated gene activation (Kemppainen et al. 1992).

### Collection, quantification, and purification of androgens from the river sediment.

Sediment was collected from the Fenholloway River on 12 June 2003 where it crosses Highway 361A, east of Perry, Florida, 3.6 km downstream of the paper mill settling ponds. A long-handle 2.8-mm mesh dip net was used to collect samples from the top 20 cm of sediment. Water was allowed to drain thoroughly from the net, leaving only pore water in the sediment sample. Five 50-mL samples of the sediment were immediately mixed with 200 mL 100% methanol (HPLC grade). Within 24 hr the sediment samples were filtered through acid-washed glass wool and vacuum-filtered through 0.8 μm cellulose nitrate membranes (Whatman, Maidstone, UK). The filtrate was passed through methanol-washed solid-phase extraction cartridges (Mega Bond Elut, 6 mL, C-18; Varian Instrument Co.). The eluant was dried under N_2_ gas. Fractions were reconstituted in methanol for HPLC fractionation and in ethanol for the androgen receptor transcription assay. Steroid quantification was based on peak area in HPLC relative to coeluting standard steroids and was represented as mean of five samples ± SE.

### Liquid chromatography–mass spectrometry of river sediment.

We used LC-MS to verify the identity of androgens in the HPLC, C-18 column fractions from the Fenholloway River sediment that induced androgen receptor–mediated transcriptional activity and were preliminarily identified based on co-migration with standards. Individual HPLC fractions were dried under N_2_ gas and reconstituted in 100% methanol for LC-MS verification on a Shimadzu VP system (Shimadzu, Kyoto, Japan). We used a 10 cm × 2.1 mm, C-8 Aquapore column (Applied Biosystems, Foster City, CA) with a 12 min linear 0–100% methanol gradient in 10 mM ammonium acetate to separate the components. Eluants were passed into an electrospray interface of a PE Sciex API III triple-quadrupole mass spectrometer (PE Sciex, Foster City, CA). Multiple reaction monitoring was used for the final comparison of unknown compounds with standards. In this procedure, the parent ion was selected with the first quadrupole and passed into the second quadrupole containing argon gas. Collision of the parent ion with the argon produced fragment ions. Monitoring of a specific parent ion by the first quadrupole and ion fragments by the third quadrupole constituted the multiple reaction monitoring method. Elution of the selected parent–fragment pair at the same chromatographic retention time as a standard confirmed the identity of the steroids.

## Results

### In situ *production of androgens from progesterone by* M. smegmatis.

**Figure 1:**
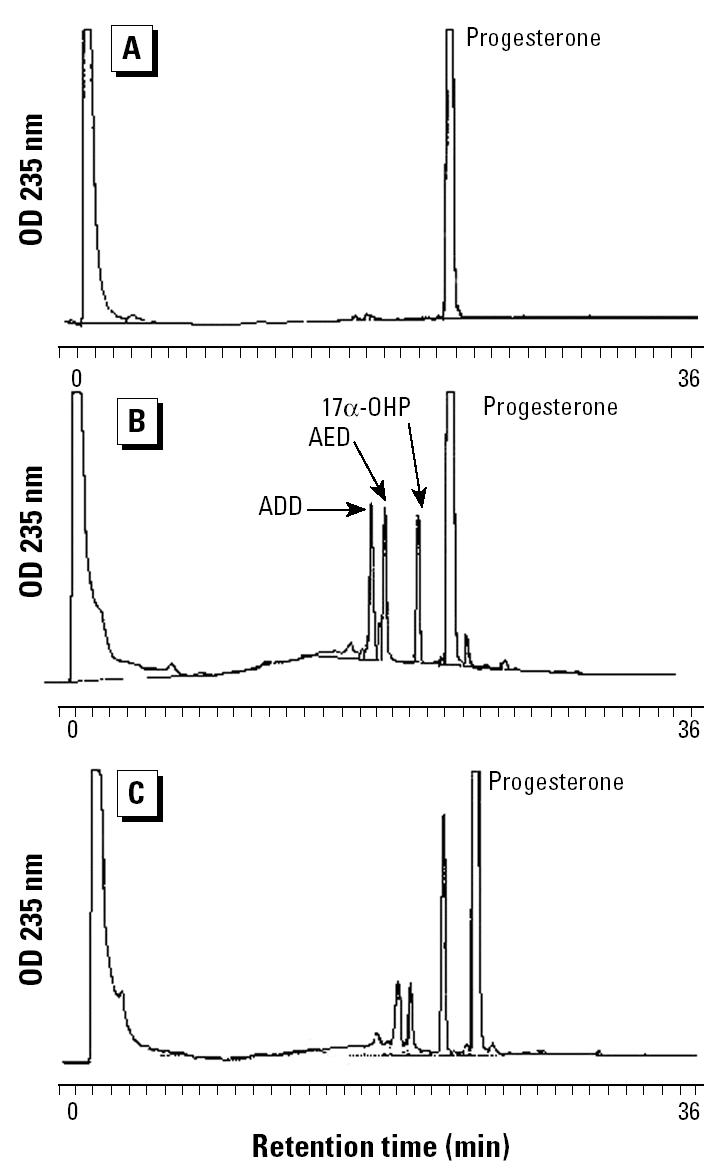
Reverse-phase, C-18, 36-min gradient HPLC separation of methanol extractions of *M. smegmatis* cultures containing progesterone (1 mM) after days 0 (*A*), 6 (*B*), and 20 (*C*) of incubation at 24–25°C. Samples were extracted in 80% methanol, vacuum filtered, and separated by C-18 solid-phase extraction. HPLC solvent A was 0.25% *O*-phosphate, and solvent B was 100% methanol with 20% methanol from 0 to 5 min, linearly increased to 100% methanol from 20 to 36 min. Detection was by absorbance (OD, optical density) at 235 nm, and the full vertical scale represents 1 absorbance unit (10^6^ μV).

**Figure 2:**
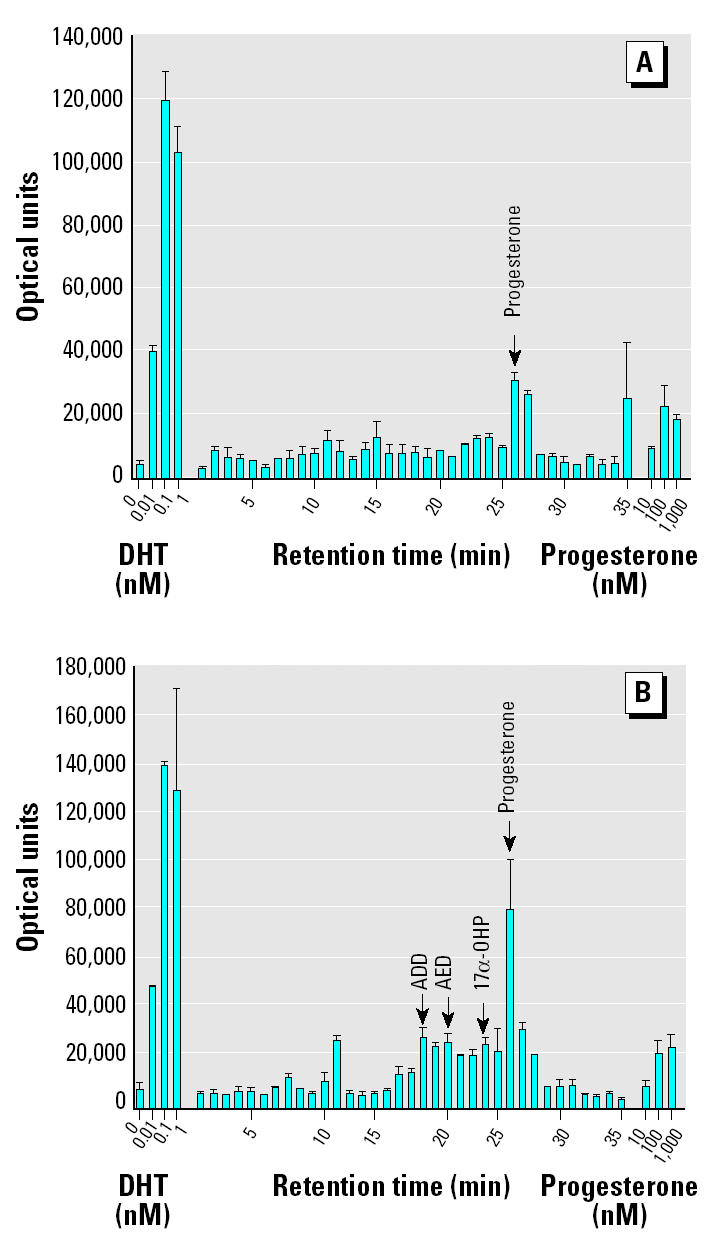
Androgen receptor–mediated transcriptional activity (mean optical units ± SE; *n* = 2) of HPLC fractions from *M. smegmatis* extractions taken at day 0 (*A*) and day 6 (*B*) of incubation. Cotransfection assays were performed in monkey kidney CV-1 cells as described in “Materials and Methods.” Luciferase activity was measured in optical units. Minutes on the abscissa refer to elution time of the HPLC separation. DHT was the positive control.

**Figure 3:**
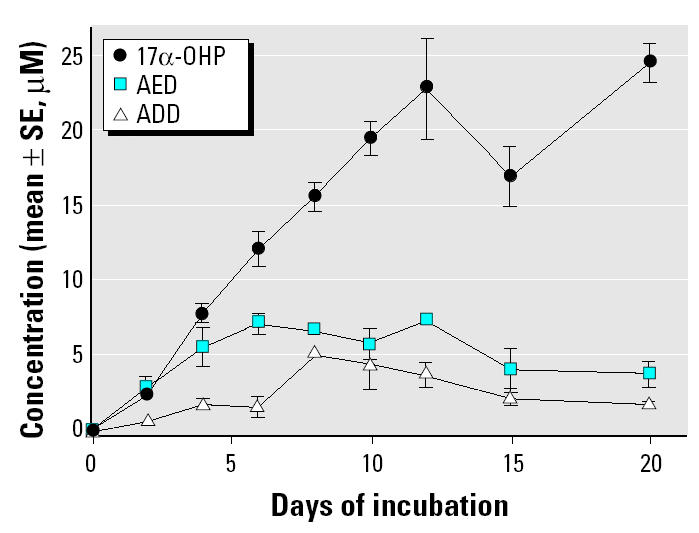
Steroidal derivatives from the biotransformation of 1 mM progesterone by *M. smegmatis* from day 0 to day 20 of incubation. See “Materials and Methods” for details of bacterial incubation, sample extraction, and HPLC fractionation. Quantification of steroid levels was based on mean peak areas ± SE (*n* = 3) compared with standard steroids.

*M. smegmatis* incubated with progesterone (1 mM) produced steroidal products that paralleled HPLC standards 17α-OHP, AED, and ADD. Figure 1 illustrates the HPLC chromatograms from 0 days (Figure 1A), 6 days (Figure 1B), and 20 days (Figure 1C) of incubation and the progressive production of 17α-OHP, AED, and ADD. Figure 2 shows the androgen receptor transcription assay for samples removed at day 0 (Figure 2A) and day 6 (Figure 2B), illustrating the accumulation of androgenic components. The sample from day 6 (Figure 2B) contained an unidentified component in the 12-min fraction that stimulated androgen receptor–mediated transcription as well as the steroids in the 19- to 24-min fractions. The 12-min fraction component did not match the elution time of any of our steroid standards and remains unidentified. Figure 3 graphically represents the production of steroids in the incubation medium over the 20-day incubation period. The concentration of 17α-OHP steadily increased from day 0 through day 12. The highest mean concentration of 17α-OHP (23.0 ± 0.7 μM) was detected on day 20. The apparent drop in concentration at day 15 may have resulted from a sampling error. The 12-, 15-, and 20-day mean values do not differ significantly from each other (analysis of variance, *p* = 0.070). AED concentrations increased from day 0 to day 6, with a maximum mean concentration of 7.2 ± 0.8 μM. AED levels remained steady until day 12 and declined thereafter. ADD concentrations were consistently lower than those of AED, except at day 8, when the mean was maximal at 5.2 ± 0.5 μM (Figure 3).

### Verification of androgens from river sediment.

**Figure 4:**
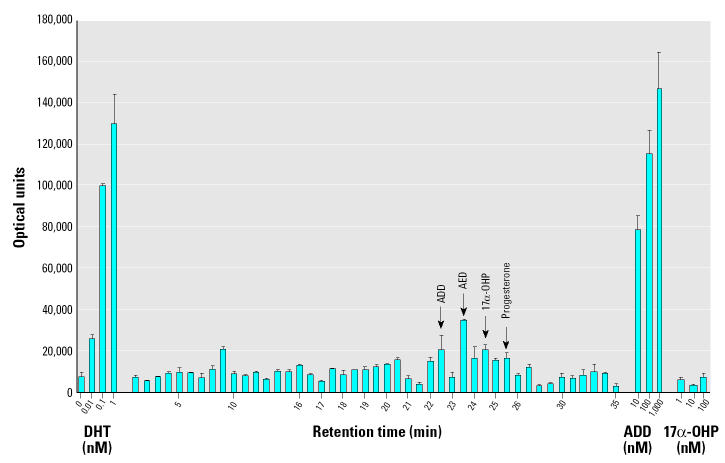
Androgen receptor–mediated transcriptional activity (mean optical units ± SE; *n* = 2) of gradient HPLC fractions from Fenholloway River sediment. Cotransfection assays were performed in monkey kidney CV-1 cells as described in “Materials and Methods.” Luciferase activity was measured in optical units. Minutes on the abscissa refer to elution time of the HPLC separation. DHT was the positive control.

After the tentative identification of AED and ADD from *M. smegmatis* incubations, sediment from the Fenholloway River was extracted to determine whether these androgens were present. Figure 4 illustrates the androgen receptor transcription assay activity induced by fractions from a gradient HPLC separation of a crude extract of sediment. From the HPLC separation and agonist activity results, fractions containing ADD (22 and 22.5 min), AED (23 min), and progesterone (25 min) were isolated. The verification of AED and progesterone by LC-MS has been previously published (Jenkins et al. 2003).

**Figure 5:**
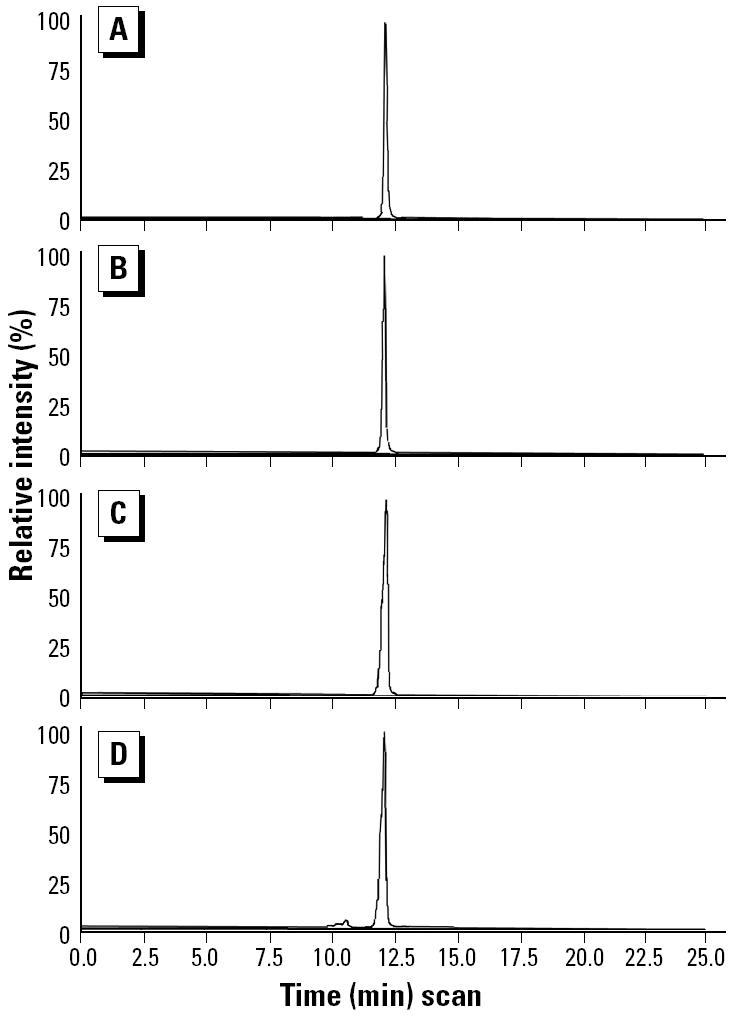
LC-MS with multiple reaction monitoring chromatograms of parent and ion fragments of ADD standard and Fenholloway River sediment sample. (*A*) 285/121 fragment ion of 100 pmol ADD standard. (*B*) 285/151 fragment ion of 100 pmol ADD standard. (*C*) 285/121 fragment ion of the 22-min HPLC fraction from Fenholloway River sediment. (*D*) 285/151 fragment of the 22-min HPLC fraction from the Fenholloway River sediment.

The principal steroid of the 22- and 22.5-min fractions was verified as ADD by LC-MS and compared with an ADD standard (Figure 5). The parent compound of the sample and standard had a molecular weight of 285 and fragmented to two ions: 285/121 and 285/151. The ratio of the parent ion and two fragment ions (285/121:285/151) was 4.12 for the standard and 4.11 for the sample.

### Steroid levels in the Fenholloway River sediment.

The concentrations (mean ± SE) of progesterone, 17α-OHP, AED, and ADD in sediment samples from the Fenholloway River were 150.3 ± 39.9, 11.1 ± 2.2, 4.0 ± 1.0, and 2.6 ± 0.4 nM, respectively. Steroids were quantified from HPLC chromatograms based on the peak area in five replications compared with standard peak areas. These values were similar to previously cited values (Jenkins et al. 2003).

### Androgen receptor transactivation by ADD and AED.

**Figure 6:**
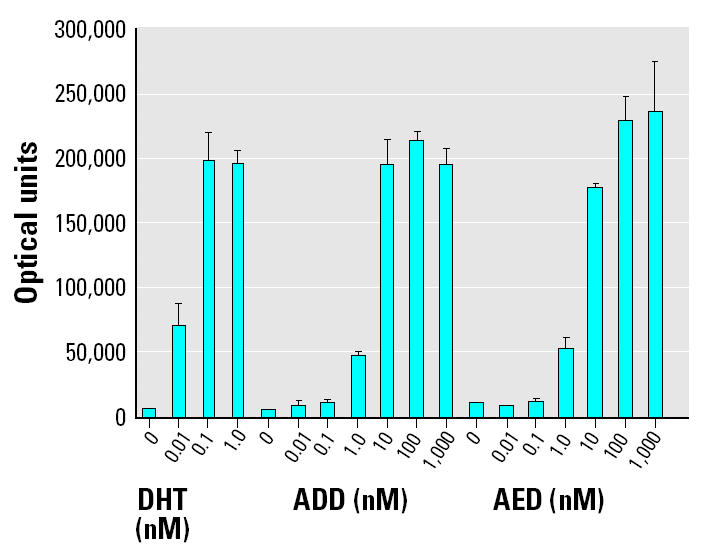
Relative effectiveness of DHT, ADD, and AED in the monkey kidney CV-1 androgen receptor–mediated transcription assay. Luciferase activity was measured in optical units, and values shown are mean ± SE.

Transcriptional activity induced by commercially purified forms of the androgenic microbial products that were verified in the sediment of the Fenholloway River was compared with DHT (Figure 6). The relative potencies of ADD and AED to act as agonists in the mammalian androgen receptor–mediated transcription assay were essentially identical. Agonist activity induced by 1 nM ADD or 1 nM AED was nearly equivalent to the response obtained using 0.01 nM DHT, indicating a 100-fold less potency of ADD and AED relative to DHT.

## Discussion

**Figure 7:**
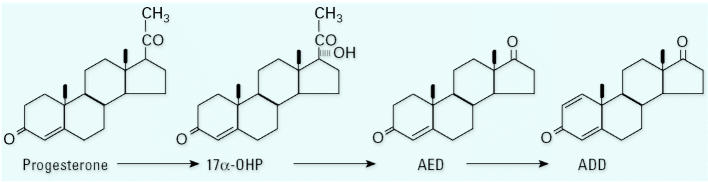
The sequence of *in vitro* production of androgens from progesterone by a culture of *M. smegmatis*.

The results of this study are consistent with our hypothesis that steroids detected in the Fenholloway River water and sediment derive from microbial biotransformation of phytosteroids in the waste stream from the wood pulping process. Nagasawa et al. (1969) showed that many genera of microorganisms, including *Arthrobacter*, *Nocardia*, *Protaminobacter*, *Serratia*, *Streptomyces*, *Mycobacterium*, and *Microbacterium*, contain the enzymes necessary to convert cholesterol and other C-17 sterols to AED and ADD. Other investigators have shown that microbial degradation of plant sterols and cholesterol can produce ADD (Marsheck et al. 1972; Roy et al. 1991). Conner et al. (1976) cultured *Mycobacterium* species with tall (pine) oil sterols and showed that ADD was the principal product. Thus, the presence of ADD in the Fenholloway River water and sediment could result from the metabolism of phytosterols by a variety of bacterial strains. By slowing the reactions using a cooler incubation temperature (25°C), we were also able to identify the intermediates. Based on the rate of accumulation of the intermediates during the reaction (progesterone > 17α-OHP > AED > ADD), the implied sequence of *in vitro* production of these androgens from progesterone by a culture of *M. smegmatis* is shown in Figure 7. Figure 3 illustrates that the pathway continues with a decline of ADD beyond day 8 of incubation, implying the presence of additional reaction steps that metabolize ADD.

Progesterone and AED were recently identified in relatively high concentrations in the sediment of the Fenholloway River at a site that received no inflows other than paper mill effluent (Jenkins et al. 2003). Peck et al. (2004) demonstrated that the sediment of rivers below sewage treatment plants in the United Kingdom are major sinks for steroidal estrogens derived from sewage. Conner et al. (1976) showed that the river sediment below a paper mill contains an abundance of phytosterols. The present study demonstrates that phytosterols have been converted to progesterone via microbial activity and progesterone to the androgenic compounds AED and ADD.

With respect to the masculinization of mosquitofish in the Fenholloway River (Howell et al. 1980), ADD could be a more important androgen than AED, which has been shown to be present in the water column and sediment of the Fenholloway River (Jenkins et al. 2001, 2003). Data in the present study indicate that AED and ADD have similar abilities to induce mammalian androgen receptor–mediated transcription. However, AED is more rapidly aromatized to estrogen. ADD has an inhibitory effect on P450 aromatase (*k_i_* = 0.32 mM; *k*_inact_ = 0.91 × 10^−3^/sec) and may irreversibly inactivate aromatase by forming enzyme–substrate covalent bonds (Covey and Hood 1982). The 1-androstene prohormones (including ADD) can be converted to 1,4-androstadien-17α-ol-3-one (boldenone). The rates of aromatization of ADD and boldenone to estrogen are about half those of the 4-androstenes (Steele et al. 1977). Because ADD is resistant to aromatization, it would be expected to have a longer and more effective androgenic half-life and thus be a more potent androgen *in vivo*.

The production of ADD by microbial degradation of phytosteroids may also have more widespread implications than its effects on aquatic wildlife near paper mills. The microbial degradation pathways seen in this study also occur in the intestine of humans and wildlife, where the native bacterial flora could provide the appropriate enzymes for steroid biosynthesis. *Escherichia coli* has been shown to degrade cholesterol to AED and ADD (Owen et al. 1978). ADD has been isolated from the feces of cattle after subcutaneous injection of progesterone (1 mg/day for 5 days) (Miller et al. 1956). Owen et al. (1978) concluded that progesterone was sequestered by the liver and converted by microbes in the intestines to ADD. It appears that dietary phytosterols may have human health benefits in terms of improving blood lipid profiles (St-Onge and Jones 2003; Vorster et al. 2003) and in reducing the risk of some cancers (Messina and Barnes 1991; Philpotts 1997). If ADD and other Δ1-androgens are generated in the human gut from the degradation of phytosterols to steroidal precursors, other effects on human health may need to be considered.
